# Effect of Grain Size on Thermophysical Properties in Twinning-Induced Plasticity Steel

**DOI:** 10.3390/ma18040890

**Published:** 2025-02-18

**Authors:** Joong-Ki Hwang

**Affiliations:** School of Mechatronics Engineering, Korea University of Technology & Education, Cheonan 31253, Republic of Korea; jkhwang@koreatech.ac.kr; Tel.: +82-41-560-1642

**Keywords:** thermophysical properties, thermal conductivity, grain size, twinning-induced plasticity steel

## Abstract

This study investigated the thermophysical properties of TWIP steel with respect to grain size. The coefficient of thermal expansion (*β*) of TWIP steel was approximately 22.4 × 10^−6^ °C^−1^, and this value was hardly affected by the grain size. Therefore the density of TWIP steel was also unaffected by grain size within the tested range. The *β* in TWIP steel was higher than that of plain carbon steels (13–15 × 10^−6^ °C^−1^) such as interstitial free (IF) steel and low-carbon steel, and stainless steels (18–21 × 10^−6^ °C^−1^) such as X10NiCrMoTiB1515 steel and 18Cr-9Ni-2.95Cu-0.58Nb-0.1C steel. The specific heat capacity (*c*_p_) increased with temperature because the major factor affecting *c*_p_ is the lattice vibrations. As the temperature increases, atomic vibrations become more active, allowing the material to store more thermal energy. Meanwhile, *c*_p_ slightly increased with increasing grain size since grain boundaries can suppress lattice vibrations and reduce the material’s ability to store thermal energy. The thermal conductivity (*k*) in TWIP steel gradually increased with temperature, consistent with the behavior observed in other high-alloy metals. *k* slightly increased with grain size, especially at lower temperatures, due to the increased grain boundary scattering of free electrons and phonons. This trend aligns with the Kapitza resistance model. While TWIP steel with refined grains exhibited higher yield and tensile strengths, this came with a decrease in total elongation and *k*. Thus, optimizing grain size to enhance both mechanical and thermal properties presents a challenge. The *k* in TWIP steel was substantially lower compared with that of plain carbon steels such as AISI 4340 steel, especially at low temperatures, due to its higher alloy content. At room temperature, the *k* of TWIP steels and plain carbon steels were approximately 13 W/m°C and 45 W/m°C, respectively. However, in higher temperature ranges where face centered cubic structures are predominant, the difference in *k* of the two steels became less pronounced. At 800 °C, for example, TWIP and plain carbon steels exhibited *k* values of approximately 24 W/m°C and 29 W/m°C, respectively.

## 1. Introduction

The demand for lightweight automobiles continues to rise, driven by the need to enhance energy efficiency and reduce environmental impact. With the increasing adoption of electric vehicles, the emphasis on lightweight design has intensified. In response, steel industry researchers are focused on developing high-strength steels to reduce the weight of cars, aiming to maintain competitiveness against alternative materials in the automotive sector. For example, dual-phase (DP) steel, high-strength low-alloy (HSLA) steel, martensitic steel, and transformation-induced plasticity (TRIP) steel have been under development for decades to reduce density and consequently fuel consumption [1]. Fundamentally, steels face challenges in competition with other materials due to their high density. Additionally, excessively increasing the strength reduces formability, making the plastic forming process more difficult. Therefore, efforts are being made to develop various materials and processes that can simultaneously improve strength and formability. One of the most practical ways to improve steel’s strength is through the adjustment of alloy compositions. Consequently, research efforts in the steel industry have increasingly centered on optimizing chemical compositions to utilize various hardening mechanisms, such as solid solution strengthening, precipitation hardening, and enhancements achieved through heat treatment. However, as alloying elements are added, steel’s thermal properties tend to decline, which can lead to challenges in manufacturing processes and performance under service conditions. TWIP (twinning-induced plasticity) steels, for example, have emerged as promising alternatives to conventional steels due to their excellent strength, ductility, toughness, and resistance to hydrogen-induced fracture [2,3,4,5,6]. The remarkable mechanical properties of TWIP steels are linked to their deformation mechanisms, such as deformation twinning and/or dynamic strain aging (DSA) [7,8,9,10,11,12,13]. To achieve a stable austenitic structure, TWIP steels require relatively high manganese content (10–25%), which may negatively affect the thermal properties in TWIP steels [14].

Generally, in steels, as the alloying content increases, thermal conductivity (*k*) decreases and the linear thermal expansion coefficient (*β*) increases. For example, comparing low-alloy plain carbon steels and high-alloy stainless steels, the *k* of plain carbon steel at room temperature is approximately 45 W/m°C, while that of stainless steel is approximately 14 W/m°C [14,15,16]. Additionally, the *β* of plain carbon steels is around 14 × 10^−6^ °C^−1^, whereas that of stainless steels is about 20 × 10^−6^ °C^−1^ [17,18,19,20]. Therefore, high alloy contents in metals can affect manufacturing processes, including plastic forming, casting, machining, and welding. For instance, *k* plays a vital role in managing temperature gradients in both manufacturing and service environments. These gradients can create thermal stress, especially during thermal cycling or high heat flux situations. Additionally, in heat treatments such as quenching, *k* limits the permissible size of the product. Thus, a detailed understanding of thermal properties is essential for designing efficient manufacturing processes for high-alloyed metals such as TWIP and stainless steels. In the case of TWIP steel, there is considerable effort to apply the steel to products by utilizing its high strain-hardening rate through the plastic forming processes [8,21]. To successfully perform the plastic forming process, it is essential to carefully consider the thermal history and/or thermal stress that occur during the deformation. One of the factors that influence the thermal history of the material during plastic deformation is its thermal properties. Therefore, it is essential to investigate the temperature-dependent changes in the thermal properties of TWIP steel. In addition, given the trend toward higher alloy content for improved in-service performance, studying thermophysical properties has become increasingly important. TWIP steel, for example, is being considered for high-temperature applications, where a deep understanding of its thermal properties is necessary to ensure reliability and safety.

Meanwhile, one limitation in commercializing TWIP steel is its relatively low yield strength (YS) despite its high tensile strength (TS) and uniform elongation [22,23,24]. To address this, several studies have investigated the influence of grain size on the mechanical properties of TWIP steels, as it is known that decreasing grain size increases YS [25,26,27,28,29,30,31]. For instance, Ueji et al. [28,29] demonstrated that YS improved and deformation twinning became more challenging as grain size decreased in Fe-31Mn-3Al-3Si steel. Similarly, Rahman et al. [32] showed an increase in YS and twinning stress with smaller grain size in Fe-15Mn-2Al-2Si-0.7C steel. Similar findings were also reported by Gutierrez-Urrutia et al. [25], Lee et al. [26], Kang et al. [27], and Shen et al. [31] using Fe-22Mn-0.6C, Fe-24Mn-4Cr-0.5C, Fe-18Mn-1.5Al-0.6C steels, and Fe-20Mn-0.6C, respectively. These studies suggest that refining grain size can effectively improve YS in TWIP steels by modifying the rate of twinning and deformation mechanisms. However, if the grain size of TWIP steel is made finer to increase its YS, there is a possibility that its thermal properties could deteriorate. While numerous studies have examined the influence of grain size on mechanical properties such as YS, TS, and elongation, few have explored its impact on thermophysical properties in TWIP steel. It is known that *k* increases and *β* decreases in general metals as the grain size increases. Grain boundaries act as scattering sources for free electrons and phonons; therefore, grain boundaries diminish *k* in materials [33]. In addition, it is known that grain boundaries have a higher *β* compared to the grains’ interior; thus, *β* decreases with grain size [34]. It has not yet been studied whether the changes in thermal properties with grain size apply directly to TWIP steel.

This study, therefore, examines how grain size influences the thermophysical properties of TWIP steel. To accomplish this, high-manganese TWIP steels with three distinct grain sizes were produced, and their mechanical properties, microstructures, and thermophysical properties were analyzed. Thermophysical properties such as *k*, thermal diffusivity (*α*), *β*, density (*ρ*), and isobaric specific heat (*c*_p_) are known to be highly temperature-dependent [35,36]. Accordingly, *α*, *β*, and *c*_p_ of TWIP steel were measured across a range of temperatures, and *ρ* and *k* were subsequently calculated based on these measurements.

## 2. Experimental Procedures

### 2.1. Materials Preparation

A 50 kg ingot of TWIP steel was fabricated via induction melting in a furnace under Ar gas, with its chemical composition provided in Table 1. During casting, a riser was used to remove the solidification shrinkage section. To reduce chemical segregation, the ingot was homogenized at 1200 °C for 3 h in a furnace. Subsequently, hot rolling and cooling were performed to break the cast structure and obtain a normalized hot rolling structure. The thickness of the ingot was reduced using several rolling mills with a reduction ratio of approximately 20% per pass, resulting in a total reduction of 84%. In other words, the 125 mm thick ingot was rolled into a 25 mm plate through several passes without intermediate reheating. The final rolling temperature, measured using a pyrometer, was approximately 950 °C. The plate was then naturally cooled in air. The ambient temperature was 21 °C. The plate was cut into four pieces to obtain samples that were heat-treated at different temperatures. Heat treatments were applied to the samples using an electric furnace under N_2_ gas for 30 min at 700 °C, 1100 °C, and 1250 °C to achieve a broad range of grain sizes. After heat treatment, the specimens were naturally air-cooled in ambient air.

### 2.2. Measurement of Microstructure and Mechanical Properties

Microstructural analysis, focusing on grain size and orientation, was conducted using electron backscatter diffraction (EBSD). To prepare samples for EBSD, specimens were sequentially ground with commercial silicon carbide papers up to 2000 grit using an automated polisher, followed by polishing with diamond pastes ranging from 1 to 3 μm. For the final preparation, a colloidal silica suspension was applied for approximately 1.2 ks. EBSD data were collected using a field-emission SEM equipped with a TexSEM Laboratories EBSD system (AMETEK, Berwyn, PA, USA), operating at an acceleration voltage of 20 kV and a tilt angle of 70°. The data were then processed with orientation imaging microscopy software (version 8). To analyze the constituent phases of the specimen with grain size, X-ray diffraction (XRD) measurements were conducted using Cu K_α_ radiation (*λ* = 0.154056 nm) at an operating voltage of 30 kV at room temperature (RT, 24 °C). The scan range was set to 35–100° with a scanning speed of 1°/min. The microstructure analysis was conducted on a specimen sectioned perpendicular to the hot rolling direction (RD). That is, the RD plane of the specimen was observed.

Cylindrical tensile specimens with a gauge diameter of 5 mm and a gauge length of 25 mm were machined parallel to RD. At RT, testing was conducted using an Instron at an initial strain rate of 10^−3^ s^−1^. Strain measurements were obtained with a mechanical extensometer.

### 2.3. Measurement of Thermophysical Properties

The *α*, *β*, and *c*_p_ of the specimen were measured with temperature and grain size. Thermomechanical analysis (TMA) was performed using a TA Instruments TMA Q400 (TA Instruments, New Castle, DE, USA) with a cylindrical specimen with a diameter of 5.0 mm and an initial length (*L*₀) of 12.0 mm to measure *β*. The test was conducted with a heating rate of 5 °C/min under an N_2_ atmosphere. Using Δ*L*/*L*_0_, *β* was calculated as follows:

(1) $$\beta (T)=\frac{∆L}{L_{0}}\frac{1}{∆T}$$

where the subscript o denotes the reference value at RT. Measurements were carried out from RT to 950 °C. The volume of a specimen (*V*) was estimated as follows:

(2) $$V={{L_{0}}^{3}(1+\frac{∆L}{L_{0}})}^{3}$$

Assuming Δ*L*/*L*_0_ is small, this equation was approximated as follows based on Taylor series expansion:

(3) $$V=V_{0}(1+3\frac{∆L}{L_{0}})$$

Then, *ρ* was obtained based on the measured *ρ*_0_ and *β* using the following equation:

(4) $$\rho =\frac{m}{V}\;\;{=\rho }_{0}\frac{1}{1+3\beta_{L}∆T}$$

where *m* represents the mass. *ρ*_0_ was measured by the Archimedes’ principle using a precision balance at RT. The specimen weights were measured both in air and in a state of immersion in distilled water.

The *c*_p_ was measured from 25 to 420 °C using a differential scanning calorimeter (DSC) with simultaneous thermal analysis (STA), Netzsch STA 449 F5 Jupiter (NETZSCH-Gerätebau GmbH, Selb, Germany). An Ar flow was maintained throughout the test, and the instrument was calibrated with a sapphire reference following baseline correction with empty pans. Detailed measurement procedures can be found in Ref. [37].

The *α* of the TWIP steel was obtained by laser flash analysis (LFA) using a Netzsch LFA 467 HT (NETZSCH-Gerätebau GmbH, Selb, Germany) with a sample in the form of a disk, measuring 10.0 mm in diameter and 2.5 mm in thickness [38]. Based on the principle originally developed by Parker et al. [39], *α* was obtained as follows:

(5) $$\alpha =0.1388\frac{l^{2}}{t_{0.5}}$$

where *l* is sample thickness and *t*_0.5_ is the time required for the surface temperature to reach half of the maximum temperature increase. Both sides of the sample were coated with graphite to improve absorption of the laser radiation, and Ar gas was used during testing. Measurements were taken at 100 °C intervals from RT to 1000 °C. Since the sample expanded with temperature, *α* was corrected as follows [40] to account for thermal expansion:

(6) $$\alpha_{true}=\alpha {\left(1+\beta ∆T\right)}^{2}\;\;\;$$

where *α*_true_ refers to the corrected *α*. Overall, k was calculated indirectly based on the measured *α* and *c*_p_, and calculated *ρ* as follows [41]:

(7) $${k\left(T\right)=\alpha_{true}(T)\rho (T)c}_{p}(T)$$

## 3. Results and Discussion

### 3.1. Microstructure and Mechanical Properties

**Figure 1:**
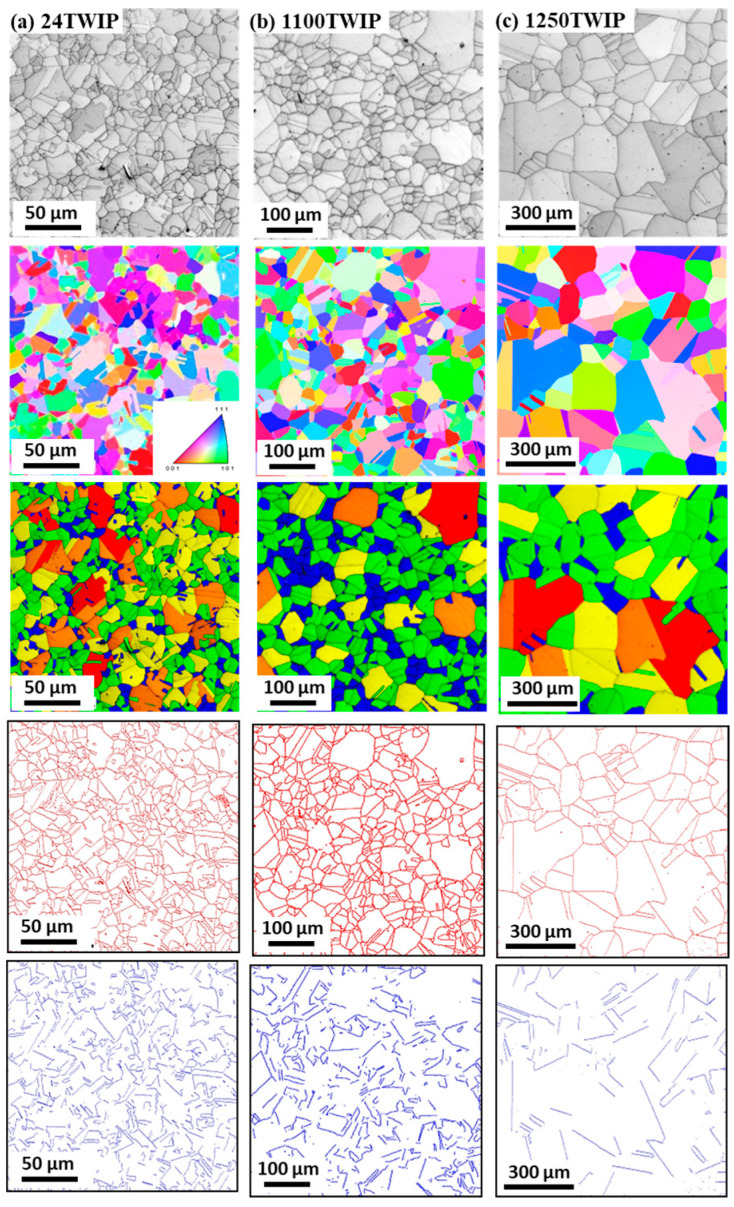
Comparison of EBSD IQ, IPF, grain shape major axis, high-angle grain boundaries (red), and twin boundaries (blue) maps of (**a**) 24 TWIP, (**b**) 1100 TWIP, and (**c**) 1250 TWIP steels.

**Figure 2:**
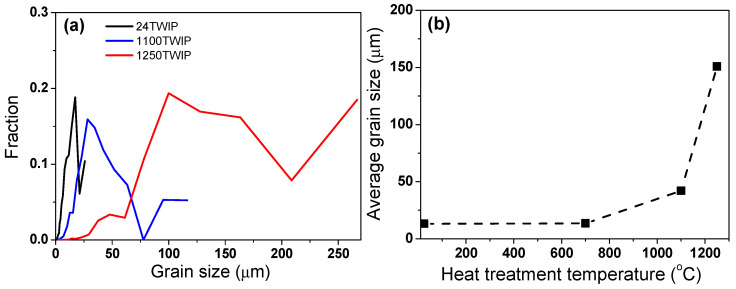
(**a**) Calculated grain size and (**b**) average grain size with heat treatment temperature.

**Figure 3:**
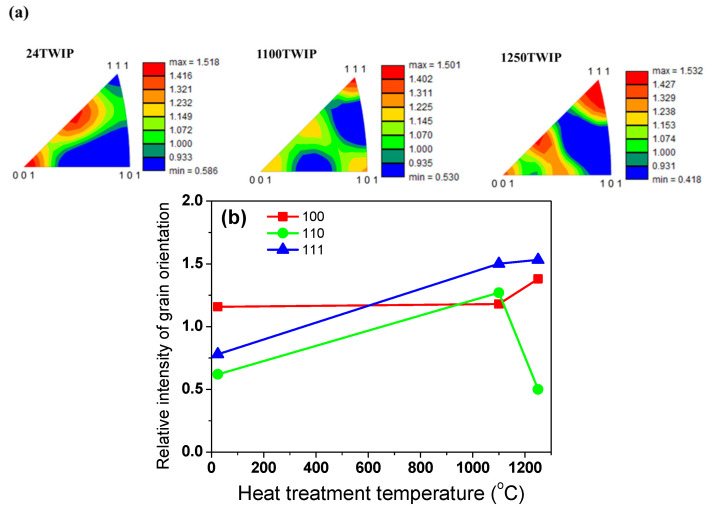
(**a**) Grain orientation and (**b**) its intensity of TWIP steels with grain size.

**Figure 4:**
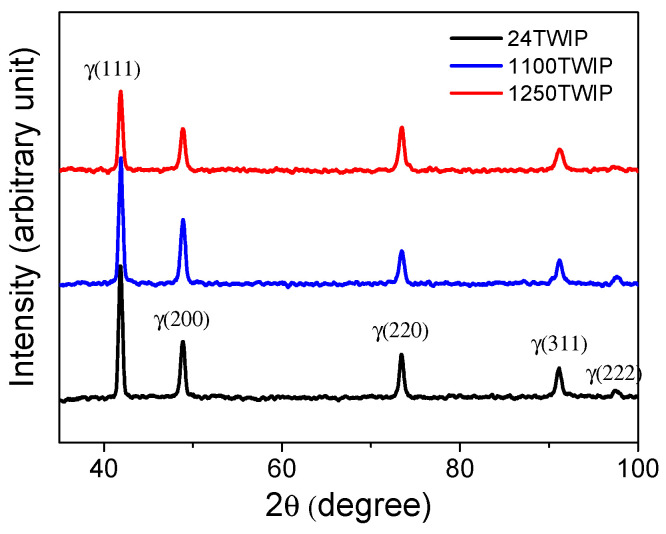
X-ray analysis of constituent phases with grain size.

Figure 1 compares the microstructures of the specimen with different heat treatment temperatures using EBSD image quality (IQ), inverse pole figure (IPF), grain shape major axis, high-angle grain boundaries, and twin boundaries maps. High-angle grain boundaries were defined as those with a misorientation angle of 15° or more and are displayed in red lines in the high-angle grain boundaries maps. Twin boundaries, characterized by a misorientation angle between 57° and 63°, are shown as blue lines in the twin boundaries maps. Accordingly, high-angle boundaries include twin boundaries. All microstructures were composed of fully recrystallized grains with a high fraction of annealing twins. The grain size, including annealing twins, increased with heat treatment temperature, as summarized in Figure 2. The specimen heat-treated at 700 °C exhibited a similar grain size to the untreated TWIP steel. Therefore, the analysis focused primarily on the untreated TWIP steel and those heat-treated at 1100 °C and 1250 °C, which are referred to as 24 TWIP, 1100 TWIP, and 1250 TWIP, respectively, for convenience. The average grain sizes of the 24 TWIP, 1100 TWIP, and 1250 TWIP were 13.2, 42.1, and 150.8 μm, respectively. The grain size of the specimen increased as the heat treatment temperature rose, which is similar to the results obtained by Kang et al. [27]. Figure 3 shows the grain orientation of the present TWIP steel with heat treatment temperature or grain size. The 24 TWIP exhibits a high level of (100) orientation, while the 1100 TWIP and 1250 TWIP show a pronounced (111) texture. However, it is difficult to conclude that a texture has developed in this steel considering the intensity of each orientation: for all specimens, the maximum intensity of the texture did not exceed 1.6. Figure 4 shows the X-ray diffraction result of the TWIP steels with grain size. Only austenitic peaks were observed regardless of grain size, meaning that they consisted of an austenite single phase. This result can also be inferred from the stacking fault energy of the present TWIP steel. Using thermodynamic models [42,43], the stacking fault energy of the steel was 27.2 mJ/m^2^. Saeed-Akbari et al. [42] showed that a stacking fault energy of 20 mJ/m^2^ is considered the upper limit for ε-martensite formation in high-manganese steels. Based on the microstructure evolution, the influences of grain shape, grain orientation, and phase need not be considered to analyze the thermophysical properties of TWIP steel with grain size in this study. Meanwhile, it should be noted that precipitates, segregations, and inclusions also affect the thermophysical properties of metallic materials [44,45]. In this study, analysis was conducted using EBSD; thus, such features could not be well detected in the EBSD analysis, which is a limitation of the present study.

**Figure 5:**
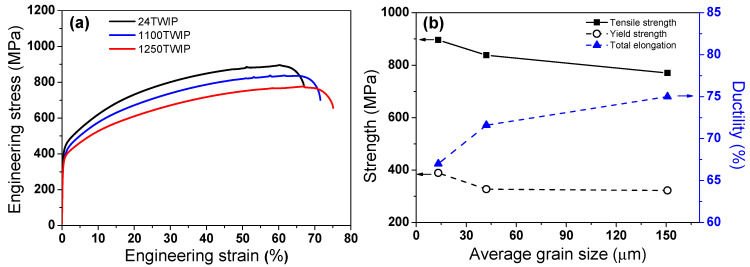
Comparison of (**a**) engineering stress-strain curves and (**b**) variations in strength and ductility of the present TWIP steel with grain size.

Figure 5a presents the engineering stress-strain curves of the TWIP steels with grain size. All three steels showed an outstanding combination of strength, ductility, and toughness. Regardless of the grain size, TS is above 700 MPa, and total elongation is over 60%, resulting in high toughness. The outstanding mechanical properties of the TWIP steels are associated with the formation of deformation twinning during tensile deformation [8,22]. Small post-necking elongation and serrations were observed, which are general characteristics of C-added high-manganese TWIP steels [22,46]. Grain refinement improved the YS and TS with a slight reduction in ductility, as shown in Figure 5b, consistent with the Hall–Petch relationship [26,47].

### 3.2. Thermal Expansion Coefficient, Density, and Specific Heat Capacity

**Figure 6:**
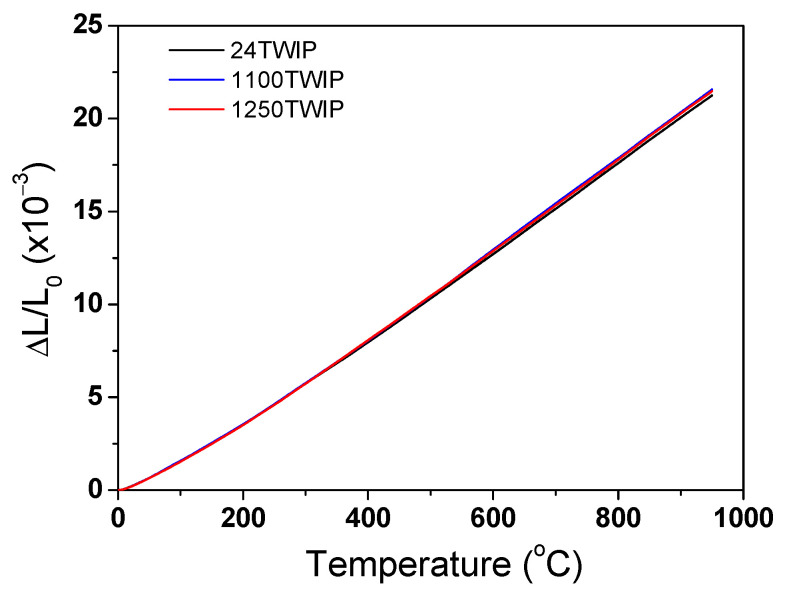
Comparison of measured thermal expansion ratio in the present TWIP steel with temperature and grain size.

**Figure 7:**
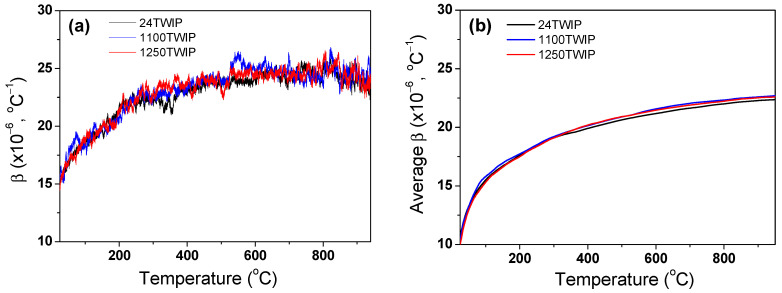
Comparison of (**a**) instantaneous and (**b**) average thermal expansion coefficients of TWIP steel with temperature and grain size.

Figure 6 compares the measured thermal expansion ratio (Δ*L*/*L*_0_) of the TWIP steel with temperature and grain size. The thermal expansion ratio of all the specimens increased with temperature and showed a similar value regardless of grain size. Figure 7a shows the comparison of variations in instantaneous *β* with temperature and grain size. The instantaneous *β* gradually increased with temperature despite slight fluctuations, exhibiting similar value regardless of grain size. It is known that the *β* of materials generally decreases with increasing grain size due to the high anharmonic atomic vibration in grain boundaries compared with the interior of the grain or lattice [34,48,49]. For example, Klam et al. [34] revealed that the *β* of the grain boundary in copper was 2.5 to 5.0 times larger than that of the grain interior. In this study, the differences in grain size between 13.2 μm and 150.8 μm were too small to produce a significant variation in *β* values with grain size, leading to similar *β* regardless of grain size. For example, Klam et al. [34] derived the dependency of grain boundary on *β* in Cu alloy with a small grain size of 17 μm and large grain size of 19 mm. In addition, Liu et al. [48] reported that *β* decreased with increasing grain size in Fe-Ni invar alloy with a grain size range of 12.5 nm–10 μm. Meanwhile, to accurately understand the effect of the grain boundary on *β*, factors such as grain boundary angles, precipitates, inclusions, and the type of grains during heat treatments and hot rolling should also be considered. In this study, only the effect of grain size was examined, which can be regarded as a limitation of this research. For practical use, the average *β* was obtained based on the measured Δ*L*/*L*_0_, as shown in Figure 7b. In this graph, the values at each temperature represent the average *β* from 25 °C to that temperature. The average *β* increased with temperature regardless of grain size. Overall, the average *β* from 25 to 900 °C in this TWIP steel was approximately 22.4 × 10^−6^ °C^−1^. It is worth mentioning that the *β* of the TWIP steels was much higher compared with that of plain carbon steels (13–15 × 10^−6^ °C^−1^), such as interstitial free (IF) steel and low-carbon steel, and slightly higher compared with that of stainless steels (18–21 × 10^−6^ °C^−1^), such as X10NiCrMoTiB1515 steel and 18Cr-9Ni-2.95Cu-0.58Nb-0.1C steel, possessing a face centered cubic (FCC) structure in Refs. [17,18,19,20]. This result is associated with their crystal structures, such as BCC and FCC. Bohemen [50] showed that the β of steels with FCC was higher than those with BCC.

**Figure 8:**
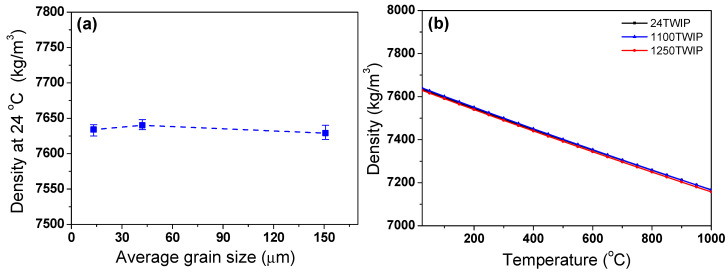
Comparison of (**a**) measured density at room temperature and (**b**) variations in calculated density of TWIP steel with grain size and temperature.

The average *β* from 25 to 900 °C was used to calculate the *ρ* of this TWIP steel. The measured *ρ*_0_ values are presented in Figure 8a. The average *ρ*_0_ was also similar to the grain size. Figure 8b shows the calculated *ρ* as a function of temperature, and it showed similar values regardless of grain size. As expected, *ρ* decreased with increasing temperature, which is consistent with the behavior of typical metals [35,51]. Meanwhile, the *ρ* of the TWIP steel was lower than that of plain carbon steels due to the high manganese and aluminum contents in this steel. The *ρ* of manganese and aluminum are approximately 7.21 and 2.70 kg/m^3^, respectively. These values are much lower than the ρ of iron, which is approximately 7.87 kg/m^3^. This low *ρ* of TWIP steels offers significant advantages as a lightweight material for automotive applications.

**Figure 9:**
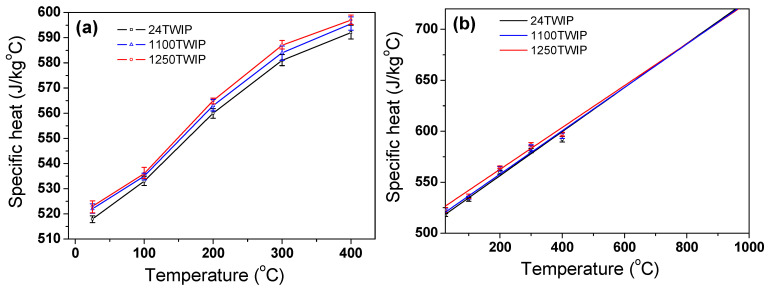
Comparison of (**a**) measured and (**b**) linear fitted specific heat of present TWIP steel with temperature.

Figure 9a compares the measured *c*_p_ of TWIP steel with temperature and grain size. Due to the limitations of the DSC equipment used in this study, *c*_p_ was measured up to 400 °C. *c*_p_ increased with temperature because the major factor affecting *c*_p_ is the lattice vibrations. In other words, as the temperature increases, atomic vibrations become more active, allowing the material to store more thermal energy, resulting in an increase in *c*_p_ [33]. Overall, *c*_p_ slightly increased with increasing grain size, which is consistent with previous research findings. Zhang et al. [52] reported that the *c*_p_ of OFHC copper increased with grain size, and Roy et al. [53] revealed that the *c*_p_ of silicone sheet increased with increasing grain size. Grain boundaries contain atoms with irregular arrangements, which can suppress lattice vibrations and reduce the material’s ability to store thermal energy, resulting in a decrease in *c*_p_. In other words, as the grain size increased, the number of grain boundaries decreased, allowing atoms to vibrate more freely, leading to an increase in *c*_p_. Since no transition in crystal structure and magnetic properties is well known in high manganese TWIP steels [8,54], *c*_p_ was expected to increase almost linearly with temperature, just like austenitic stainless steels [55] and high-alloyed tool steels [56]. Consequently, *c*_p_ above 400 °C was estimated based on a linear fit of the measured *c*_p_ values, as illustrated in Figure 9b. At lower temperatures, *c*_p_ varied depending on grain size, but the variation in *c*_p_ due to grain size decreases as the temperature increases. At low temperatures, phonon scattering at grain boundaries limits the material’s ability to store thermal energy. In contrast, as the temperature increases, atoms vibrate more freely, making the effect of grain size less significant in determining *c*_p_.

### 3.3. Thermal Diffusivity and Conductivity

**Figure 10:**
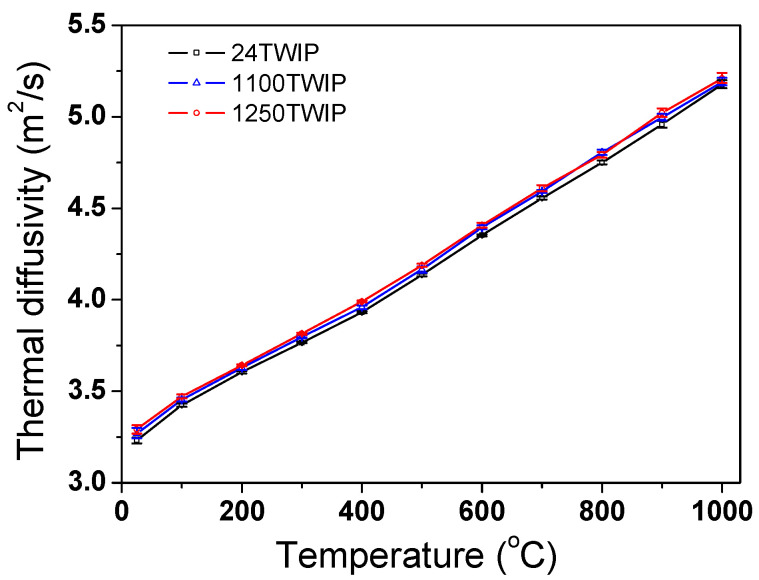
Comparison of measured thermal diffusivity of present TWIP steel as functions of temperature and grain size.

**Figure 11:**
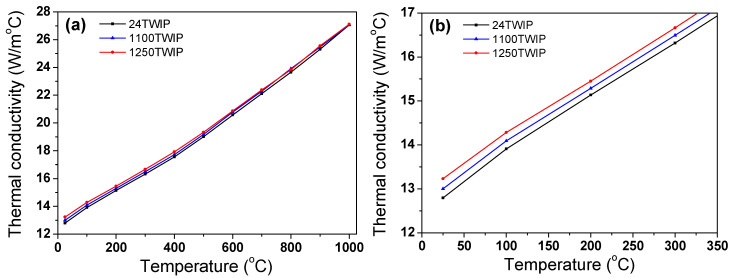

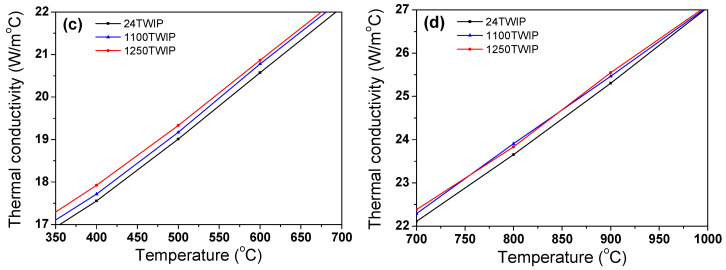
Comparison of thermal conductivity of TWIP steel with grain size and temperature: (**a**) full temperature scale, (**b**) low-temperature region, (**c**) mid-temperature region, and (**d**) high-temperature region.

Figure 10 shows the comparison of the measured *α* of the steel with temperature and grain size. It is observed that *α* increased with temperature and grain size. Figure 11 shows the calculated *k* with temperature and grain size. The *k* of TWIP steel increased as temperature increased regardless of grain size, consistent with findings for stainless steels with an FCC structure [16], Ni-Co-based superalloys [57], and high-entropy alloys [58]. Energy is mainly transported by free electrons and phonons in metal; thus, *k* can be expressed as a sum of heat transfer via free electrons (*k*_e_) and phonons (*k*_p_) [33,59]. Generally, in metals, *k*_e_ has a greater impact on *k* than *k*_p_ due to the higher velocity of free electrons compared to phonons [60]. In more detail, *k* is influenced by both the quantity of carriers and the mobility of free electrons. In high-alloyed metals, as temperature rises, the effect of the free electron carriers outweighs that of their mobility. This is because free electron mobility is reduced due to high levels of impurities in high-alloys [61]. That is, at lower temperatures, electron scattering is significant due to the high alloy content, rendering the temperature-dependent mobility of electrons less impactful as temperature rises. As a result, *k*_e_ increases with temperature owing to the larger number of free electron carriers, although their mobility decreased with increasing temperature. Overall, the *k* of TWIP steels rose with increasing temperature.

**Figure 12:**
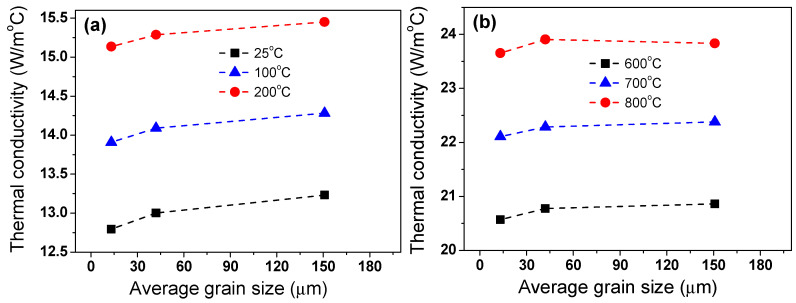
Comparison of variations in thermal conductivity of TWIP steel with grain size at (**a**) low temperatures of 25, 100, and 200 °C and (**b**) high temperatures of 600, 700, and 800 °C.

The different behaviors of *k* were also observed with grain size. In the TWIP steel, *k* increased with grain size because *c*_p_ and *α* increased with grain size despite the similar *ρ* with grain size. To make it easier to visualize the behavior of *k* according to grain size, *k* was presented with grain size at the fixed temperature, as represented in Figure 12. Various factors, including dislocations, solute atoms, grain boundaries, and precipitates, act as scattering sources for both free electrons and phonons, which can typically diminish k in materials [33]. For example, the *k* of materials increases as the grain size increases because grain boundaries are scattering centers that impede the movement of free electrons and phonons [62,63,64,65,66]. Tong et al. [64] reported that the *k* of as-cast pure magnesium increased with grain size, which is in agreement with the results of Oh et al. [62]. In ceramics, *k* increased with increasing grain size [65,66]. Liu et al. [63] revealed that the *k* of nanostructured ZnO decreased as the grain boundary angle between two grains increased using molecular dynamics simulation. Meanwhile, *k* varied with grain size in the low-temperature region. However, in the high-temperature region, changes in grain size have little effect on *k* in TWIP steel, indicating that the impact of grain size on *k* was more pronounced at lower temperatures. At low temperatures, phonon scattering at grain boundaries is the dominant factor governing heat transfer. However, as the temperature increases, phonon-phonon collisions or scattering become more frequent [33], reducing the relative influence of grain size. In other words, while larger grain sizes enhance *k* at low temperatures, phonon-phonon interactions become the primary source of thermal resistance at high temperatures, diminishing the effect of grain size. Additionally, at high temperatures, increased electron scattering makes electron-electron scattering and electron-phonon scattering the primary factors limiting *k* rather than grain size. As a result, the influence of grain size on *k* becomes relatively less significant at higher temperatures.

**Figure 13:**
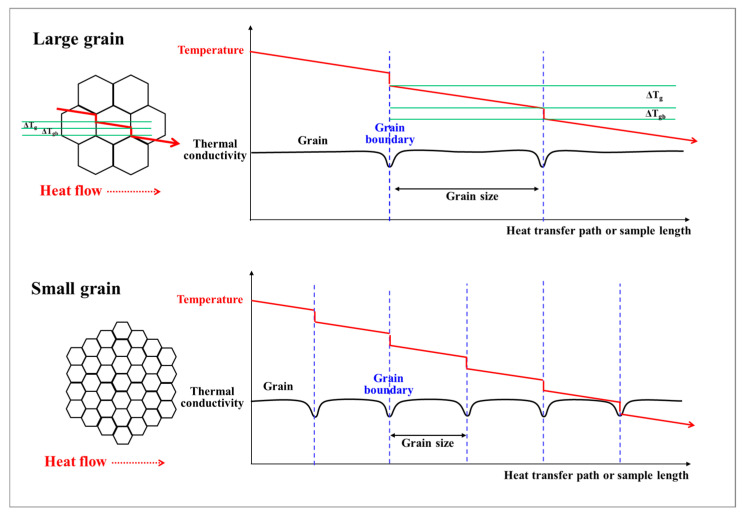
Schematic description of one-dimensional temperature profiles along polycrystalline materials with large and small grain boundaries.

**Figure 14:**
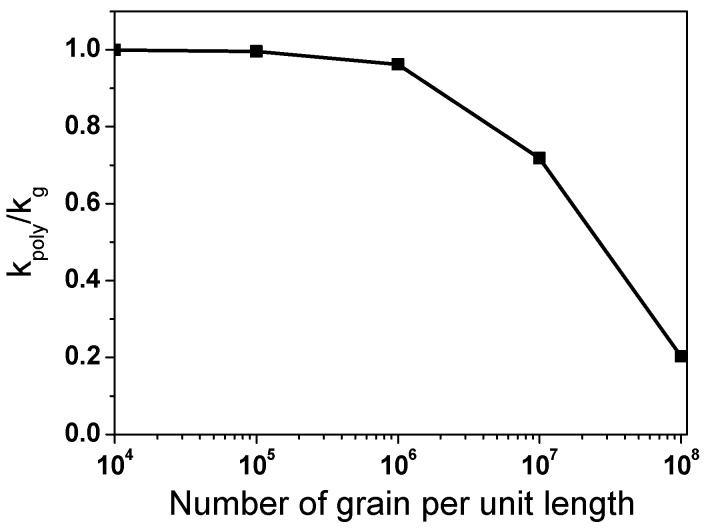
Variations in calculated ratio of thermal conductivity in polycrystalline to single crystal of TWIP steel with number of grains per unit length.

Figure 13 describes the schematic of one-dimensional temperature profiles along polycrystalline materials with large and small grain boundaries. It is well known that the atomic arrangement within 2–3 atomic distances is chaotic in grain boundaries. In other words, the interface between two neighboring grains has different orientations. The localized chaotic region in grain boundaries can scatter free electrons and phonons, leading to a localized degradation in *k*, as illustrated in Figure 13. The reduction of *k* at the grain boundaries, compared with the grain interior, leads to a significant temperature gradient in this region. That is, the thermal gradient steepens near the grain boundaries, and the temperature difference across one grain (Δ*T*) is expressed as follows:

(8) $$∆T={∆T}_{g}+{∆T}_{gb}$$

where (Δ*T*_g_) and (Δ*T*_gb_) indicate the temperature difference in the grain interior and the temperature difference in the grain boundary, as shown in Figure 13, respectively. The shape of the temperature drop (Δ*T*_gb_) or temperature discontinuity in grain boundaries can be modeled as an interface thermal resistance at the grain boundary (*R*_gb_), which is known as Kapitza resistance [67]. Although heat transfer occurs solely through phonons due to the absence of free electrons in ceramic materials, for simple analysis of *k* with grain size, *k* in polycrystalline materials (*k*_poly_) can be represented as follows, based on the brick layer model applied to ceramic materials [68].

(9) $$\frac{1}{k_{poly}}=\frac{1}{k_{g}}+nR_{gb}$$

where *k*_g_ refers to *k* in the grain interior, and *n* is the number of grain boundaries per unit length along the heat transfer path. The second term on the right side of the above equation represents the effect of grain size. As the grain size decreases, n increases, resulting in a decrease in *k*_poly_ in materials. Figure 14 shows the variations in the calculated *k*_poly_/*k*_g_ of TWIP steel with the number of grains per unit length. The *R*_gb_ was calculated to be approximately 3.9 × 10^−8^ m^2^ °CW^−1^ based on Equation (9) and the experimental results of this study. The *k*_poly_/*k*_g_ gradually decreased with increasing *n*. Although this graph was not entirely accurate due to numerous assumptions in Equation (9), it is effective for understanding the trend of *k* with respect to grain size in materials. Meanwhile, as the temperature increased, the grain size dependence of k decreased, as shown in Figure 11 and Figure 12, due to the reduction in *R*_gb_ with rising temperature [69].

**Figure 15:**
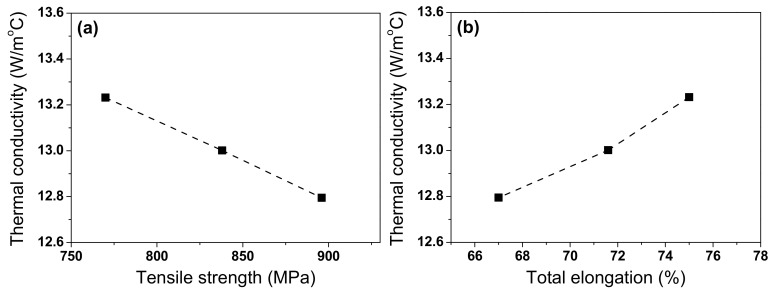
Relationships between (**a**) tensile strength and thermal conductivity, and (**b**) total elongation and thermal conductivity of TWIP steel at room temperature.

To find the relationships between mechanical properties and *k* in TWIP steel, Figure 15 shows the relationships between tensile strength and thermal conductivity, and total elongation and thermal conductivity of TWIP steel at RT. As the grain size increased, TE and *k* increased, but TS decreased. This result indicated that finding the optimal grain size that enhances both mechanical and thermal properties was challenging in TWIP steel. Meanwhile, it should be noted that the microstructure of the experimentally cast and rolled specimens in this study may not be identical to that of actual hot-rolled steels. As a result, the thermophysical properties of the steel in this experiment may also differ from those manufactured in industrial hot rolling processes. For example, the hot rolling speed and reduction ratio of the experimentally produced steel may differ from those of the steel fabricated in industry.

Finally, it is necessary to compare the thermal conductivity of the TWIP steel obtained in this study with that of commonly used carbon steel. The *k* in TWIP steel is substantially lower compared with that of plain carbon steels, such as AISI 4340 steel and 26NiCrMoV10 steel [15,36,70]. This discrepancy in *k* is particularly evident at lower temperatures, primarily due to the relatively high mobility of free electrons in plain carbon steels at low temperatures, resulting from their lower alloy content compared to TWIP steel. For instance, at RT, the *k* values for plain carbon steels were about 45 W/m°C, while the present TWIP steel showed a much lower *k*, around 13 W/m°C. However, in higher temperature ranges where an FCC structure predominates, the *k* values in TWIP steels and plain carbon steels become more comparable as the mobility of free electrons decreases with increasing temperature, due to the enhanced phonon scattering with temperature [61]. By 800 °C, for example, TWIP and plain carbon steels exhibited *k* values of approximately 24 W/m°C and 29 W/m°C, respectively.

## 4. Conclusions

Since TWIP steels maintain an austenitic single-phase structure even at high temperatures, it is necessary to evaluate both their thermophysical and mechanical properties to explore their potential as high-temperature materials. While various studies focus on refining the grain size to improve the yield strength of TWIP steels, investigating the changes in thermophysical properties associated with grain size remains limited. Therefore, the present study examined the thermophysical properties in TWIP steel with grain size and temperature to better understand its behavior in relation to thermal and mechanical performance. Key findings are as follows:

1. The *β* and density in TWIP steel showed minimal variation with grain size, suggesting that the grain size does not significantly affect these properties within the tested range. The average *β* from 25 to 900 °C in this TWIP steel was approximately 22.4 × 10^−6^ °C^−1^. Meanwhile, the *β* of the TWIP steels was higher compared with that of plain carbon steels (13–15 × 10^−6^ °C^−1^), such as interstitial free (IF) steel and low-carbon steel, and slightly higher compared with that of stainless steels (18–21 × 10^−6^ °C^−1^), such as X10NiCrMoTiB1515 steel and 18Cr-9Ni-2.95Cu-0.58Nb-0.1C steel, both possessing an FCC structure.
2. The *c*_p_ of TWIP steel increased with temperature because the major factor affecting *c*_p_ is the lattice vibrations. As the temperature increases, atomic vibrations become more active, allowing the material to store more thermal energy, resulting in an increase in *c*_p_. Meanwhile, *c*_p_ slightly increased with increasing grain size. Grain boundaries contain atoms with irregular arrangements, which can suppress lattice vibrations and reduce the material’s ability to store thermal energy, resulting in a decrease in *c*_p_.
3. The *k* of TWIP steel increased as temperature increased, consistent with the behavior observed in other high-alloy metals. In addition, *k* slightly increased with grain size, especially at lower temperatures, due to the increased grain boundary scattering of free electrons and phonons. This trend aligns with the Kapitza resistance model, which emphasizes grain boundaries as critical scattering centers for heat transfer in materials. However, in the high-temperature region, changes in grain size have little effect on *k* in TWIP steel, indicating that the impact of grain size on *k* was more pronounced at lower temperatures.
4. While TWIP steel with refined grains exhibited higher yield and tensile strengths, this came with a decrease in total elongation and *k*. Thus, optimizing grain size to enhance both mechanical and thermal properties presents a challenge.
5. The *k* in TWIP steel was substantially lower compared with that of carbon steels, such as AISI 4340 steel and 26NiCrMoV10 steel, especially at low temperatures, due to its higher alloy content. At room temperature, the *k* of TWIP steels and plain carbon steels were approximately 13 W/m°C and 45 W/m°C, respectively. In contrast, in higher temperature ranges where FCC structures are predominant, the difference in *k* between TWIP steels and plain carbon steels diminished, primarily due to reduced electron mobility from enhanced phonon scattering. At 800 °C, for example, TWIP and plain carbon steels exhibited *k* values of approximately 24 W/m°C and 29 W/m°C, respectively.

## Figures and Tables

**Table 1:** Chemical compositions of present TWIP steel.

Compositions (wt.%)					
C	Mn	Al	P	S	Fe
0.66	17.10	1.75	<0.01	<0.01	Balance

## Data Availability

The original contributions presented in this study are included in the article. Further inquiries can be directed to the corresponding author.
